# A curated collection of transcriptome datasets to investigate the molecular mechanisms of immunoglobulin E-mediated atopic diseases

**DOI:** 10.1093/database/baz066

**Published:** 2019-07-10

**Authors:** Susie S Y Huang, Fatima Al Ali, Sabri Boughorbel, Mohammed Toufiq, Damien Chaussabel, Mathieu Garand

**Affiliations:** Sidra Medicine, Al Gharrafa Street Ar-Rayyan, Doha, Qatar

## Abstract

Prevalence of allergies has reached ~20% of population in developed countries and sensitization rate to one or more allergens among school age children are approaching 50%. However, the combination of the complexity of atopic allergy susceptibility/development and environmental factors has made identification of gene biomarkers challenging. The amount of publicly accessible transcriptomic data presents an unprecedented opportunity for mechanistic discoveries and validation of complex disease signatures across studies. However, this necessitates structured methodologies and visual tools for the interpretation of results. Here, we present a curated collection of transcriptomic datasets relevant to immunoglobin E-mediated atopic diseases (ranging from allergies to primary immunodeficiencies). Thirty-three datasets from the Gene Expression Omnibus, encompassing 1860 transcriptome profiles, were made available on the Gene Expression Browser (GXB), an online and open-source web application that allows for the query, visualization and annotation of metadata. The thematic compositions, disease categories, sample number and platforms of the collection are described. Ranked gene lists and sample grouping are used to facilitate data visualization/interpretation and are available online via GXB (http://ige.gxbsidra.org/dm3/geneBrowser/list). Dataset validation using associated publications showed good concordance in GXB gene expression trend and fold-change.

## Introduction

Allergic disease is highly prevalent and currently reaches ~20% of the populations in developed nations and with sensitization rate to one or more allergens among school age children approaching 40%–50% (1). Although the generation of allergic responses is well understood, the early sensitization steps and factors contributing to the development of immunoglobin E (IgE)-mediated diseases remain unclear. IgE is the major mediator of atopic response in humans. Atopy is defined by the American Academy of Allergy, Asthma and Immunology as the genetic tendency to develop allergic diseases such as allergic rhinitis, asthma and atopic dermatitis (eczema) and is typically associated with heightened immune responses to common allergens, especially inhaled allergens and food allergens. However, not all encounters with a potential allergen will lead to sensitization. Similarly, not all sensitizations will result in a symptomatic allergic response even in atopic individuals.

The effect of IgE spans across multiple systems. In the circulation system, IgE increases flow and permeability of the blood vessels, fluid and protein in tissues, as well as flow to the lymph nodes. In the airway, IgE decreases air conduct diameter, increases mucus congestion and can induce blockage. In the gastrointestinal tract, IgE increases fluid secretion, peristalsis and expulsion diarrhea. The immunoglobin has also been suggested to play a role in the defense against parasite infection and as a general gate keeper for any foreign materials entering the body (2, 3). Ultimately, IgE is involved in the normal spectrum of reaction to expulse foreign material from the body, hence protecting by elimination. Nevertheless, oversensitization, which is developed by unknown mechanism(s), can lead to imbalance and pathology in affected individuals.

Two main groups of immune signals initiate the production of IgE in response to an antigen: (i) the signals that drive the differentiation of CD4 naive T cell to T helper type 2 (Th2) cells and (ii) the cytokines and co-stimulatory molecules secreted by Th2 cells, which subsequently promote T follicular helper cells-induced immunoglobulin B cells class-switch toward IgE production. Antigen characteristics, such as concentration and localization of the encounter (i.e. tissue, mucosa, circulation), can also affect Th2 cell induction. IL-4, IL-13 and STAT6 are key mediators of Th2 responses and IgM class switch to IgE. IL-4 secretion and mast cell CD40 surface expression also contribute to the IgE class switch to IgE at the site of allergic reaction.

Systemic levels of IgE alone is not a sufficient indicator for allergy risk (4). Peripheral blood IgE level can increase upon sensitization, but not reliable enough for deducing a diagnosis of allergy or allergen type (5). Concentration, binding strength or affinity, specificity and portion of specific IgE to total IgE are all factors in translating a humoral IgE response into a clinical symptom (6). However, genetic component exists in allergic disease. Studies have demonstrated strong heritable components of allergic diseases and atopy, estimated at 33%–76% (7, 8). Genetic components of food allergy and asthma have also been reviewed (9–12). Although single susceptibility genes have been identified for certain allergies (13–16), most genome-wide association studies reported multiple loci of susceptibility or gene regions to a wide range of allergy development (17–19). Environmental factors further complicate allergic susceptibility—the hygiene hypothesis of allergic susceptibility has received much attention in the last few decades (20). Other exposure-related factors (e.g. diet, pollution, tobacco smoke) are also likely to have contributed to the increasing susceptibility to allergic diseases in developed nations (1).

IgE may also be implicated in some primary immunodeficiency diseases (PIDs). For CD40 ligand and CD40 deficiency, circulating IgE+ plasma cells are absent (21). For others, IgE plasma levels are elevated: DOCK8 deficiency, autosomal dominant hyper-IgE syndrome (AD-HIES) Job’s syndrome, Comel-Netherton syndrome, PGM3 deficiency, IPEX (immune dysregulation, polyendocrinopathy, enteropathy X-linked) and Tyk2 deficiency. Large phenotypic heterogeneity is observed among these PIDs; however, the underlying mechanisms are not completely understood. This knowledge gap mirrors our limited understanding of the involved gene products mediating phenotypes of atopic diseases.

**Figure 1:**
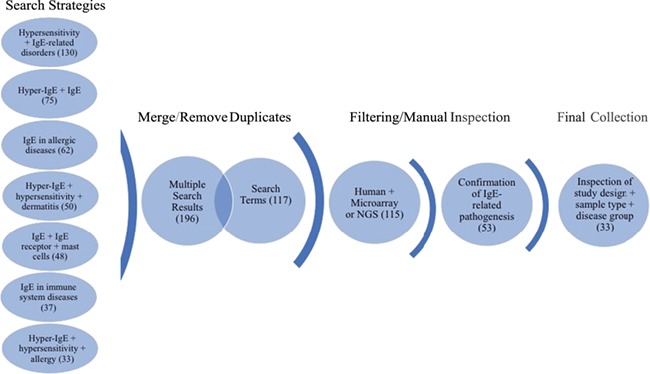
**Workflow of search strategies for datasets retrieval from the GEO database.** Datasets and search terms obtained from the seven independent search strategies were merged and with duplicates removed. The results were then filtered for human studies and microarray or NGS planforms. The studies were manually confirmed for IgE-related pathogenesis and only datasets with appropriate study design, sample type and disease groups were kept. The final collection consists of 33 datasets. Brackets indicate the number of datasets obtained after each search step.

There is a crucial need to pursue genetic markers of an atopic constitution amidst growing concern over the increasing abundance of IgE atopy and that current treatments remain mostly palliative. As both environmental interaction and genetic predisposition form the determinant of allergy development, extrapolating potential gene markers of allergy atopy from a broad range of studies with associated clinical data is highly relevant, particularly given the high variations of the disease clinical phenotypes. With the advances in high-throughput platforms for transcriptomic analyses, a large number of gene expression datasets is regularly deposited on publicly accessible repositories such as the NCBI Gene Expression Omnibus (GEO). GEO currently hosts over 90 000 datasets comprising over 2 million samples (https://www.ncbi.nlm.nih.gov/geo/) and presents an enormous opportunity for data mining across multiple studies.

The web-based Gene Expression Browser (GXB) is an open-source interface that allows for custom compilation of selected datasets (i.e. of interest to users) and facilitates visualization of gene expression data. GXB has been previously described (22) and used to generate a number of data notes (23–28). As well, GXB is a useful tool for novel gene/function discovery (29) and in system reanalysis approaches (30).

A search strategy was implemented to identify GEO datasets relevant to IgE-related atopic disease, including PIDs, and the datasets uploaded to the GXB platform online. The associated study metadata, such as detailed gene information, relevant literature, study design and sample information, were also uploaded to facilitate in-depth interpretation. In creating this collection of datasets, we aimed to provide a resource that will facilitate risk prediction and interception of allergic diseases. The datasets were retrieved from publicly available GEO series and selected by relevance to IgE and atopic diseases and filtered by analysis platform and species. As previously demonstrated, data mining and reanalysis/reinterpretation of large and publicly available dataset is a promising avenue (31) to elucidate complicated diseases such as IgE-related atopy.

## Methods

### Justification of data selection and filter

The focus of the GEO dataset selection was primarily on whether or not the dataset involved (i) IgE production or function or (ii) if the pathology studied implicated directly or indirectly IgE production or function. The dataset curation workflow is shown in Figure 1. In brief, we ensured a broad search approach by conducting seven independent search strategies. The independent search results (435) were merged using the “Merge collection” function available on `My NCBI’ (https://www.ncbi.nlm.nih.gov/myncbi/) and compiled with the results from a second search using an assembly of all the terms used in the first sets of strategies. After removal of duplicates, the two queries generated 196 and 117 datasets, respectively. The combined results were then manually filtered to restrict datasets to human sample, expression profiling by microarrays and next generation sequencing (NGS) (115) and relevance to IgE-related atopic diseases (53). Out of the remaining 53 datasets, study description, design and sample type for each dataset were inspected, with 33 datasets in the final curated collection. The following criteria were deemed very important: clear description of tissue type, comparisons between patient vs healthy or stimulated vs unstimulated samples and disease category implicating IgE. Furthermore, datasets/studies that are indirectly relevant to IgE-mediated atopic disease, such as gene expression in B cells after tonsillectomy, were also included as they were deemed valuable to (i) the discovery of putative novel gene-disease association, (ii) to improving our knowledge of adaptive immunity and/or (iii) to increasing our knowledge about factors affecting IgE production. A descriptive summary of the final dataset collection can be found in Table 1.

**Table 1:** Descriptive summary of the dataset collection

	Title	GEO ID	Platform name	Platform GEO accession number (GPL)	Disease/treatment/therapy	Sample type	Number of sample	Expt design^a^	Default group comparison^b^	Pubmed ID^c^	Trend validation	FC validation	Comments for validation
1	Allergen-specific immunotherapy modulates the balance of circulating Tfh and Tfr cells	GSE87399	Illumina HiSeq 2500	GPL16791	Allergy	PBMC/T cells	17	*In vitro*	Low allergic vs High allergic	28506846	CXCR5, FOXP3		Good validation
2	Comparison of two sets of microarray experi ments to define allergic asthma expression pattern	GSE41649	Affymetrix Human Genome U133A Array	GPL96	Allergic asthma	Bronchial biopsy	8	*Ex vivo*	Allergic asthma vs Healthy	1984284		SERPINB2, CX3CR1, C7	Strong validation
3	Distinct epithelial gene expression phenotypes in childhood respiratory allergy—disease state	GSE19190	Affymetrix Human Gene 1.0 ST Array [transcript(gene) version]	GPL6244	Rhinitis, allergy, asthma	Human nasal epithelium cells	38	*In vitro*	Rhinitis vs Healthy	22005912			Validated with stimulation
4	Distinct epithelial gene expression phenotypes in childhood respiratory allergy—Stimulation	GSE19190	Affymetrix Human Gene 1.0 ST Array [transcript(gene) version	GPL6244	Rhinitis, allergy, asthma	Human nasal epithelium cells	21	*In vitro*	IL4 vs Control	22005912		DHX58, PRIC285, STARD5	Good validaton
5	Effect of intraderma immunotherapy (IDIT) injections on gene expression profiles of activated T cells derived from skin biopsy explants	GSE72324	Illumina HumanHT-12 V4.0	GPL10558	Grass pollen allergy	T cells	15	*In vitro*	IDIT vs Control	27773851		TNFSF8, TNIP3, HDAC1	Strong validation
6	Expression data for human epithelium from subjects with atopic dermatitis, psoriasis and nonatopic controls	GSE26952	Sentrix HumanRef-8 Expression BeadChip	GPL2700	Atopic dermatitis, psoriasis	Epidermis	16	*Ex vivo*	Atopic dermatitis vs Control	21163515		GJA1, TGM1, OCLN	Strong validation
7	Expression data from irritable bowel syndrome (IBS) patients before and after treatment	GSE14842	Affymetrix Human Genome U133 Plus 2.0 Array	GPL570	Diarrhea IBS	Epidermis	14	*Ex vivo*	Oral cromoglycate vs Control	N/A			No associated publication
8	Functional classes of bronchial mucosa genes that are differentially expressed in asthma	GSE15823	Affymetrix Human Genome U95 Version 2 Array	GPL8300	Asthma	Bronchial biopsy	12	*Ex vivo*	Allergic asthma vs Healthy	15038835		SFRP1, Alox15, LDB1	Strong validation
9	Gene expression analysis related to olive pollen allergy	GSE37157	Affymetrix Human Genome U133 Plus 2.0 Array	GPL570	Olive pollen allergy	PBMC	28	*Ex vivo*	Polination asymptomatic vs Polinaztion non-allergic	23830385			No associated publication
10	Gene expression changes in early phase of venom immunotherapy	GSE92866	Affymetrix Human Gene Expression Array [Brainarray ENTREZG Version 20]	GPL22841	Venom allergy	Blood	59	*Ex vivo*	Beekeeper vs Control	N/A	CD99, SDC1	GATA3, FoxP3	No associated publication, but some articles mentioned up-regulated genes that matches with GXB data
11	Gene expression pattern of alveolar macrophages of allergic asthmatics in comparison with control subjects	GSE22528	Affymetrix Human Genome U133A Array	GPL96	Asthma	Alveolar macrophage	10	*Ex vivo*	Asthma vs. Control	19913588		CCR1, HSPD1, SERPINH1	Strong validation
12	Gene expression patterns in house dust mite (HDM) stimulated CD4 T cells and IgG to IgE ratios	GSE70760	Affymetrix Human Gene 1.0 ST Array [hugene10st_Hs_ENTREZG_19.0.0]	GPL20171	HDM	T cells	90	*In vitro*	HDM vs Control	26518094	IL4,IL13,IL17RB		Data not available or reported differently in associated publication
13	Gene expression patterns in PBMC associated with asthma exacerbation attack	GSE19301	Affymetrix Human Genome U133A Array	GPL96	Asthma	PBMC	685	*Ex vivo*	Exacerbation vs Quiet	21779351			Data reported differently
14	Gene expression profile of patients with moderate and severe chronic spontaneous urticaria	GSE72542	Agilent-039494 SurePrint G3 Human GE v2 8x60K Microarray 039381 (Feature Number version)	GPL16699	Chronic spontaneous urticaria	Skin/whole blood	61	*Ex vivo*	Patient wheal vs Healthy	28407332	FOSB, S100A9, ADAMTS4		Data not available or reported differently in associated publication
15	Gene expression profiling of patients with allergy to latex and/or vegetable food	GSE13619	Affymetrix Human HG-Focus Target Array	GPL201	Latex/plant-derived food allergy	PBMC	21	*Ex vivo*	Latex fruit vs Healthy	N/A	IFNG, STAT4, IL10RA		No associated publication, but some articles mentioned up-regulated genes that matches with GXB data
16	Genome-wide expression analysis demonstrates a dominant role of TLR4 for activation of human phagocytes by the alarmin MRP8	GSE56681	Affymetrix Human Genome U133 Plus 2.0 Array	GPL96 GPL570	Alarmins myeloid-related protein 8 and 14 signaling	Monocytes	19	*In vitro*	MRP8 vs Control	25505274		TRIP10, NFBK1, RNASE6	Good Validation
17	Genome-wide expression profiling of B Lymphocytes reveals IL4R increase in allergic asthma	GSE52742	Illumina HiSeq 2000	GPL11154	HDM	B cells	6	*In viro*	Allergy vs Control	24975796		IL4R, TCL1A, SESTD1	Strong validation
18	Human basophil expression profiles–Atopic vs Non-atopic	GSE64639	Illumina HumanHT-12 V4.0	GPL10558	Healthy subjet	Pheripheral blood	16	*In vitro*	Atopic vs Non-atopic	25962139			Data not available or reported differently in associated publication
19	Identification of IL-21-induced STAT3 dependent genes in human B cells	GSE51587	Affymetrix Human Gene 1.0 ST Array [transcript (gene) version]	GPL6244	Job’s syndrome	B cells	14	*In vitro*	AD-HIES CD40L vs Control CD40L	24159173		PRDM1, IL2RA, SGK1	Gene expression values not available in associated publication
20	Illumina Bead expression array data from Human IgE+ and IgG+ B cell subsets	GSE99948	Illumina HumanHT-12 V4.0	GPL10558	Tonsillectomy/Heathly subject	B cells	24	*In vitro*	Plasma Blast IgE+ vs B Cell IgE+	N/A	CD99, SDC1		No associated publication, but some articles mentioned up-regulated genes that matches with GXB data
21	Influence of olive pollen stimuli on the geneexpression profile in healthy controls and allergic patients	GSE54522	Affymetrix Human Genome U133 Plus 2.0 Array	GPL570	Olive pollen allergy	PBMC	46	*In vitro*	Allergic basal vs Control basal	25553522			Data not available or reported differently in associated publication
22	Integrated genomic and prospective clinical studies show the importance of modular pleiotropy for disease susceptibility, diagnosis and treatment (dataset 1)	GSE44956	Agilent-028004 SurePrint G3 Human GE 8x60K Microarray (Probe Name version)	GPL14550	Seasonal allergic rhinitis	T cells	48	*In vitro*	Low allergen GCR vs Low diluent	24571673			Data not available or reported differently in associated publication
23	Lack of allergy of timothy grass pollen is not a passive phenomenon but associated with allergen specific modulation of immune reactivity	GSE70050	Illumina HiSeq 2500	GPL16791	Pollen allergy	PBMC/T cells	76	*In vitro*	Allergic_IL5 vs NonAllergic_IL5	4846575			Discrepancy in sample names between GEO and SOFT files. Reported group comparison could not be replicated
24	Leukotriene E4 is a full functional agonist for human cysteinyl leukotriene type 1 receptor—Cell Comparison	GSE75603	Affymetrix Human Gene 1.0 ST Array [transcript (gene) version]	GPL6244	Leukotriene E4 response	Mast cells	6	*In vitro*	LUVA vs LAD2	26830450			Validated with stimulation
25	Leukotriene E4 is a full functional agonist for human cysteinyl leukotriene type 1 receptor—Stimulation	GSE75603	Affymetrix Human Gene 1.0 ST Array [transcript (gene) version]	GPL6244	Leukotriene E4 response	Mast cells	9	*In vitro*	LTD4 vs Control	26830450		STX3, GGR3, NFKBID	Good validaton
26	Neutrophil and PBMC gene expression data from Job’s Syndrome—Neutrophils	GSE8507	Affymetrix Human Genome U133 Plus 2.0 Array	GPL570	Job’s syndrome	Neutrophils	90	*In vitro*	HIES control 3 vs Healthy control 3	17881745			Validated with PBMC
27	Neutrophil and PBMC gene expression data from Job’s Syndrome - PBMC	GSE8507	Affymetrix Human Genome U133 Plus 2.0 Array	GPL570	Job’s syndrome	PBMC	51	*In vitro*	HIES control 0 vs Healthy control 0	17881745		CD151, CLEC12A, CoL6A2	Good validaton
28	Novel mediators of eicosanoid and epithelial nitric oxide production in asthma	GSE13785	Affymetrix Human Genome U133 Plus 2.0 Array	GPL96 + GPL570	Asthma	Sputum	22	*Ex vivo*	EIB pos healthy pre vs EIB neg pre	20052409		TGM2, THBS1, CD24	Good validaton
29	Progressive activation of Th2/Th22 characterizes acute and chronic atopic dermatitis	GSE36842	Affymetrix Human Genome U133 Plus 2.0 Array	GPL570	Atopic dermatitis	Skin	39	*Ex vivo*	Chronic lesion vs Normal	22951056		PI3, S100A8, S100A7	Strong validation
30	The effect of a dexamethasone and a FK506 on the induction of chemokines in human mast cells	GSE15174	Affymetrix Human Genome U133 Plus 2.0 Array	GPL570	Allergy	Mast cells	5	*In vitro*	AlgE vs DMSO	19454720		CCL1, CCL2, CCL4	Good validaton
31	Transcriptional profiling of egg allergy and relationship to disease phenotype	GSE88796	Illumina HumanHT-12 V4.0	GPL10558	Egg allergy	PBMC	132	*In vitro*	Allergic control vs Healthy control	27 788 149		CEACAM1, CLC, CCL17	Good validaton
32	Transcriptome profiling of (i) nasal polyp derived human IL17RB positive and negative T-helper cells and (ii) T-helper cells from normal nasal mucosa and matched peripheral blood	GSE70898 GSE70900	Illumina HumanHT-12V4.0	GPL10558	Chronic rhinosinusitis with nasal polyposis	T cells	24	*In vitro*	P Nasal Neg Act vs P Nasal Neg Rest	26684290		PCID2, VPS13C, GPR87	Strong validation
33	Upper airway gene expression is an effective surrogate biomarker for Th2-driven inflammation in the lower airway	GSE41861	Affymetrix Human Genome U133 Plus 2.0 Array	GPL570	Allergic asthma	Human nasal epithelium/ Bronchial biopsies	138	*Ex vivo*	Asthma Severe_N vs Healthy Control_N	N/A			No associated publication

^a^N/A = Not available.

^b^Meaning, the ranklist displayed by default in GXB for the dataset.

^c^ *Ex vivo*: Experimentation or measurements done in or on tissue from an organism in an external environment with minimal alteration of natural conditions; e.g. purification of specific cell types. *In vitro*: Experimentation or measurements done in or on altered tissue from an organism in an altered external environment; e.g. stimulation of cultured bilological specimen.

### The web-based GXB platform: a visualization tool for gene expression data

GXB is a valuable tool for training researchers about the reductionist investigative approaches (31). The creation of a web-based GXB platform was previously described in detail (22). In brief, the GXB is a simple interactive interface designed for visualization of large quantities of heterogenous data (Supplementary Figure 1). The platform allows for customizable data plots with overlapping metadata information, changeable sample order, as well as generation of sharable mini URLs that encapsulate information about the display settings in use. The dataset navigation page allows for quick identification of datasets of interest, either through filtering using predefined lists or via query terms. The user has access to multiple functionalities within the GXB graphic interface. In brief, the data-viewing interface enables interactive browsing and graphic representation of large-scale data. This interpretable format displays ranked gene lists and expression results. The interface also allows for user flexibility in terms of changing how the gene list is ranked, the method used for ranking, sample grouping (i.e. disease type), sample sorting (i.e. gender or age) and view type (i.e. bar or chart). The end user can browse through the datasets, format graph for a selected gene within a dataset and export data (i.e. annotation, FC, signal, groups) as is. The original GEO data and annotation are accessible from the GXB interface (Downloads tabs) as well as from their GEO page (links provided on Study tab). The associated source code and R scripts are publicly available at https://github.com/BenaroyaResearch/gxbrowser and https://github.com/BenaroyaResearch/gxrscripts, respectively. A detailed description of the GXB platform usage can be found under the Data Browsing and Visualization Interface section in the Supplementary Information.

### Construction of the dataset collection on GXB

The selected datasets were downloaded from GEO in SOFT file format and uploaded (http://ige.gxbsidra.org/dm3/geneBrowser/list) via the GXB interface (accessed by navigating the Tools menu located in the top-right corner of the GXB webpage; Tools/Chips Loaded/Upload Expression Data). The SOFT files contain metadata and normalized signal intensity data, generated by methods indicated by the author(s). These SOFT files can be analyzed directly for differentially expressed genes; thus, no additional processing was required. Using the `Sample Set Annotation Tool’ of the GXB interface, the datasets were annotated according to the information provided on GEO. The raw signal data type of the dataset (e.g. raw signal, log_2_ transformed and etc.) are also specified for each GEO entry. The default data display is in linear scale (see further details below in `Presentation of datasets’). When necessary, the sample annotation file (which is part of the SOFT file) were edited in order to add group information; as it was important to identify groups in order to compute fold-change (FC). For each dataset, individual samples were grouped based on relevant study variables. Three datasets (GSE19190, GSE75603 and GSE8507) were split via GXB’s `Sample Set Annotation Tool/Group Sets’ interface to provide a more meaningful group comparison (these datasets share the same GSE number in Table 1). Genes were then ranked based on FCs of the specified two-group comparison. All the information annotated and presented on the GXB is assembled in a SQL database (Data citation 1).

### Data availability

The curated datasets collected that have been described in this data note were assembled from the public repository NCBI GEO website: http://www.ncbi.nlm.nih.gov/gds/. In this study, we cited the GEO accession number of each dataset and the raw signal and annotation files are made available for download from the GXB web application (http://ige.gxbsidra.org/dm3/geneBrowser/list).

## Results

### Description of datasets

After applying the filtering strategy as previously described (Figure 1), we curated 33 datasets encompassing 1860 transcriptome profiles relevant to IgE-related atopic diseases. Detailed information on each dataset is presented in Table 1 and the summary of the data collection is presented in an aggregation of pie charts (Supplementary Figure 2).

**Figure 2:**
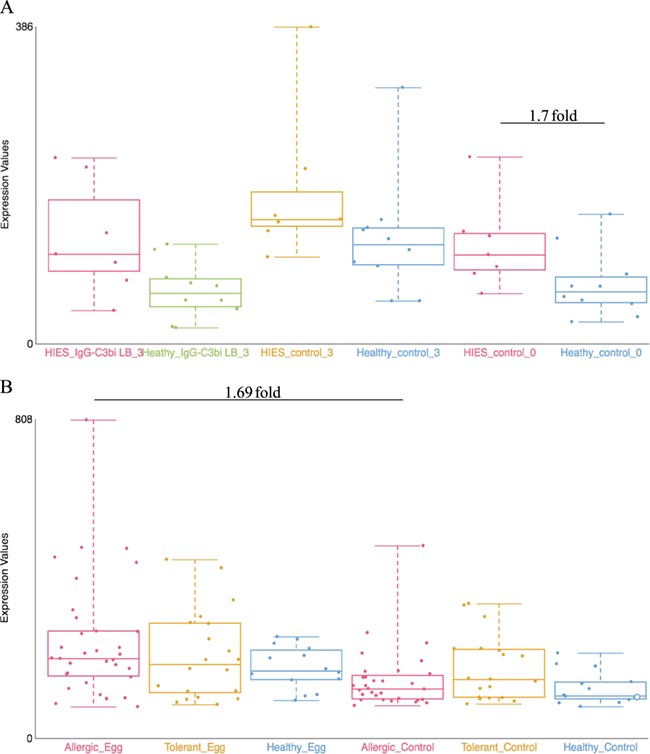
**Examples of dataset validation**. Differentially expressed genes from two datasets were compared with the results presented in the respective publications. (A) When comparing between Job’s syndrome patients (HIES_control_0) and healthy controls (Healthy_control_0), the mean fold-change of CD151 was 1.7 on the GXB (GSE8507-PBMC). The reported value in Holland *et al*. (2007) (39) was 2.0. (B) When comparing between PBMC samples from egg allergic patients (Allergic_Egg) and allergic egg-tolerant controls (Allergic_Control), the mean fold-change of CEACAM1 was 1.69 on the GXB (GSE88796). Kosoy *et al*. (2016) (40) reported a mean fold-change of 1.6 for the same gene. The overall trends of gene expression are conserved between GXB and published data.

**Figure 3:**
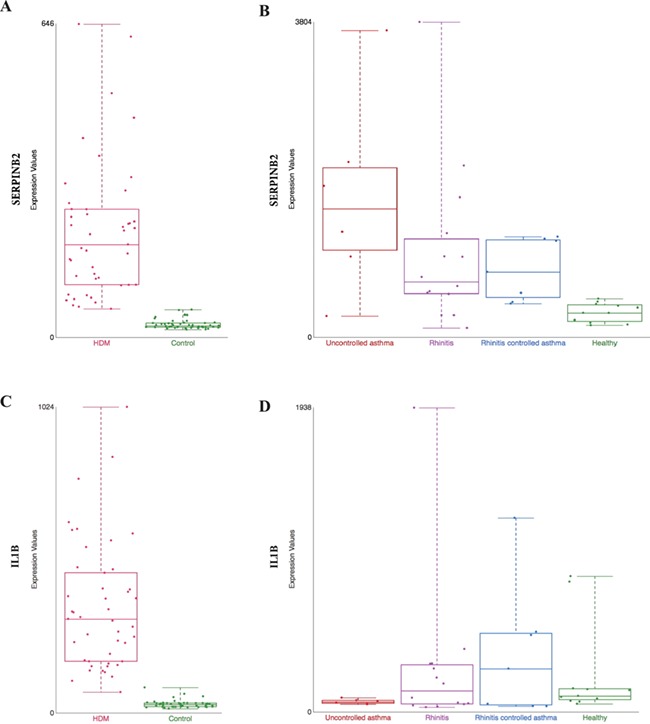
Comparison of gene expression profile in different tissues from HDM-sensitized individuals. Gene expression data from two datasets present in our collection are shown: (i) gene expression patterns in HDM-stimulated CD4 T cells and IgG to IgE ratios—GSE70760, and (ii) distinct epithelial gene expression phenotypes in childhood respiratory allergy—GSE19190—disease state. SERPINB2 (A and B) and IL1β (C and D) gene expression. HDM = house dust mite; Control = Healthy = healthy individuals; and Uncontrolled asthma = individuals with rhinitis and uncontrolled asthma (further definition can be found in the original study description (33)).

The data collection includes a wide range of studies, sample types, as well as diseases. In total, three RNAseq and 12 different microarray platforms are represented, with the majority in the latter being Affymetrix Human Array chips (various version). Twenty datasets were generated from *in vitro* studies and 13 from *ex vivo* studies. Nine sample types are represented, with the most abundant being peripheral blood mononuclear cell (PBMC) (*n* = 9) and airway epithelial cells (*n* = 5). Sample size of each dataset ranged from 5 to 628, with most studies having 10–50 samples. The dataset collection covered seven main disease categories, including allergy, asthma, healthy responses, HIES, dermatitis, atopic irritable bowel syndrome (IBS) and urticaria. In the majority of the studies, comparisons are made between patients and health controls (*n* = 19), followed by stimulation (*n* = 6) and time course (*n* = 5).

### Presentation of datasets

On the graphic interface of GXB, genes expression values are displayed in linear scale. The original signal data type can be found under the `Info’ tab of the individual datasets. The associated metadata are available under the `Samples’ tab and this information can be used for graphical overlaying (via `Overlays’ dropdown menu). The FCs are also displayed in linear scale (see Supplemental Information 2 for a detailed example of FC calculation). For FC analysis, each subject/sample are grouped according to the experimental design (experimental variable vs controls) and/or, if available, as per the corresponding publications. It is also possible to rank the genes based on expression difference by selecting `Advanced’ in the `Rank Lists’ dropdown menu of the graphic interface. This display option can be more robust than FC when low expression is observed in one group.

Additional annotation features can be used to query specific gene list from the individual datasets. Using the `Rank Lists’ dropdown function, users can select either pathways or diseases from gene list category to selectively display the FCs of the desired gene sets. These annotated gene lists come from the KEGG database (32) or are constituents of the immune-relevant genes or of genes associated with known disease signatures. The pathways option covers five gene lists, including cytokine and chemokine receptors, cytokine and chemokine ligands, T-cell signaling, B-cell signaling and antigen presenting cell processing. The nine diseases gene lists covers asthma, allograph rejection, tolerance associated genes, rheumatoid arthritis, graft vs host, type one diabetes, primary immunodeficiency, systemic lupus erythematosus and auto-immune thyroid.

It is important to note that integration and reanalysis of the datasets is not the intent of this collection. Therefore, a more meaningful use of this collection lies in the comparison of FC expression for a gene across multiple relevant datasets (i.e. reductionist approach (31)). To illustrate the cross-project view function, we presented the FC of CD40 in two datasets, GSE56681 and GSE44956, that were queried using the default cut-off of 2.0 FC (Supplementary Figure 3). The default pairwise rank comparison for each dataset and the associated FC are shown below the dataset titles. A detailed description of the cross-study features can be found in the Data Browsing and Visualization Interface section in the Supplementary Information.

### Dataset validation

Quality assessment of the datasets was performed by looking for key gene expression, i.e. marker genes, and highly differentiated genes as indicated in the associated publications. Table 1 includes the type of validation, the genes used, as well as the strength of the validation and the associated comments. Certain datasets were split in two to facilitate comparisons (GSE19190, GSE75603 and GSE8507); in these cases, validation is indicated for one of the two datasets as indicated in Table 1. Data validation was achieved for most datasets except for 7, due to having no publications (GSE14842, GSE37157 and GSE41861) or incomparable units (i.e. z-score) and/or absence of FC information (GSE64639, GSE54522 and GSE44956). One dataset (GSE70050) has a discrepancy on sample names between GEO information and SOFT files; thus, the group comparison reported in the associated publication could not be replicated in the GXB. Trend validations were done on five datasets that had no linked publications (GSE13619 and GSE99948) or had incomparable units to GXB (GSE87399, GSE70760, GSE72542). But when possible, literature values were used for additional validation of the trend (i.e. GSE92688and GSE99948). Examples of the validation results are presented in Figure 2.

## Discussion

### Potential application of the dataset collection

To demonstrate the potential use of the dataset collection, gene expression profile of different tissues for house dust mite (HDM) allergy were compared. Nasal epithelial (GSE19190) and PBMC-derived CD4 T cells (GSE70760) gene expression profiles (detailed in Table 1) were compared. In both datasets, genes associated with the Th2 pathway/axis were increased in HDM-sensitized patients compared against healthy controls (33, 34); illustrating that Th2 response may results in the symptomatic phase. This is further supported by the IgE levels and sIgG/sIgE ratio in the same study (34).

SERPINB2, a gene coding for the inhibitor of plasminogen activator PAI-2, is markedly upregulated in patients in both datasets, hence tissues (Figure 3A from GSE70760 and Figure 3B from GSE19190). This is consistent with previous association of asthma severity and biomarker panel including SERPINB2 from PBMC (35) and correlation of SERPINB2 expression in respiratory epithelial cells with atopic asthma severity (36). SERPINB2 has been reported to have a role in the interleukin-12-mediated signaling pathway; evidence from mice showed that SERPINB2 regulates IFNg production, causing down regulation of Th1 cytokines in macrophages (37).

**Figure 4:**
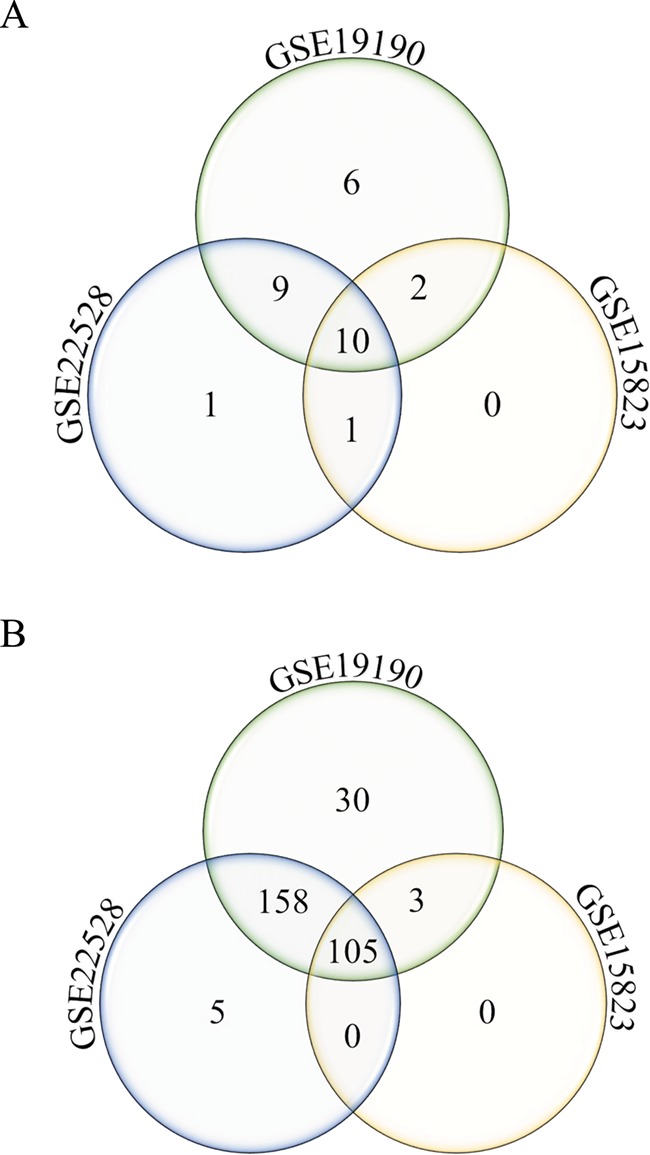
**Annotated gene list function.** Number of unique and shared differentially expressed genes from the disease asthma (A) and pathways-cytokine and chemokine receptors (B) gene lists in allergic patients and healthy controls from three different studies (GSE22528, GSE19190 and GSE15823) are shown.

The expression profiles of SERPINB2 in both tissues suggest an important role of the gene as a first line regulator of immune response, perhaps by preventing excessive Th1 response. Interestingly, IFNg was not decreased in PBMC-derived T cells, but interferon-gamma receptor 1 (IFNGR1) was, suggesting that in T cells, SERPINB2 may exert its role on the expression of the receptor rather than on IFNg itself. In Schroder *et al*. (2010) (37), stimulation of SERPINB2 −/− cells with antiCD40/IFNg resulted in greater Th1 cytokine production, thus supporting the idea that SERPINB2 affects IFNGR.

However, certain genes are found to be differently expressed between nasal epithelium or PBMC-derived T cells. An explanation is that the activation of Th1 immune response may be different in the target tissue and peripheral circulation. For example, interleukin 1 beta (IL1B), a Th1 promoting cytokine is found to be increased in CD4+ T cells in allergic patients (Figure 3C, from GSE70760), but in the nasal epithelium, the gene is not upregulated in patients with severe case of allergic rhinitis (`uncontrol asthma’) compared against healthy control (Figure 3D, from GSE19190). Further hypothesis-generating comparisons can be made by comparing the top differentially expressed genes in both datasets as listed in Supplementary Table 1.

The annotated feature can be used to query disease or pathway-specific gene lists and be applied to cross-study comparison. As an example, two specific gene lists were queried from three datasets (GSE22528, GSE15823 and GSE19190), comparing asthma or allergic and asthmatic patients to healthy controls, using (i) disease asthma and (Figure 4A) and (ii) pathway-cytokine and chemokine receptors (Figure 4B). Number of unique and shared differentially expressed genes among the three studies can be identified. In this example, 30 cytokine and chemokine receptor genes (Figure 4B) were uniquely identified to the allergic asthma disease type (GSE19190). This interesting pool of genes may potentially be used to investigate the molecular mechanisms of the co-occurrence of allergy and asthma.

## Conclusion

The dataset collection may be useful for exploring specific gene signatures in response to natural antigen/allergen exposure (i.e. allergic patients vs control) and delineate the major genetic drivers associated with increased level of IgE (i.e. cellular responses to specific antigen/allergen). Furthermore, comparative investigation of similarities and differences in expression of genes between datasets can highlight key mechanistic differences of immune signaling and/or provide insights for tissue-targeted intervention. In compiling the present dataset collection, we hope to offer a resource that may improve accessibility of public omics data to researchers in this field.

## Data citation

1. IgE_GXB_Database *Figshare*http://doi.org/10.6084/m9.figshare.7176851 (2018).

## Author contributions

MG and SSYH contributed to conceptualization. MG, SSYH and FA contributed to data curation and validation. MG and SSYH led, and FA supported, investigation and visualization. MG and SSYH performed formal analyses. SB and MT contributed to the maintenance of software. MG and SSYH contributed writing – original draft, methodology and project administration. MG and SSYH led, and FA, SB, MT and DC supported, writing–review and editing. MG lead and SSYH supported supervision. DC contributed funding acquisition and resources. The contributor’s roles listed above (underlined) follow the Contributor Roles Taxonomy (CRediT) described in Nature Communication 2014 (38) and managed by The Consortia Advancing Standards in Research Administration Information (CASRAI) (https://casrai.org/credit/).

## Supplementary Material

SUPPLEMENTAL_INFORMATION_baz066

## Notes

**Database URL**: http://ige.gxbsidra.org/dm3/geneBrowser/list

## References

[1] Pawankar R., Holgate S., Canonica G.W. et al. (2013) *WAO White Book on Allergy 2013 Update*. World Health Organization, Malwaukee, Wisconsin, USA.

[2] Nyan O.A., Walraven G.E., Banya W.A. et al. (2001) Atopy, intestinal helminth infection and total serum IgE in rural and urban adult Gambian communities. Clin. Exp. Allergy, 31, 1672–1678. 11696042 10.1046/j.1365-2222.2001.00987.x

[3] Gurish M.F., Bryce P.J., Tao H. et al. (2004) IgE enhances parasite clearance and regulates mast cell responses in mice infected with Trichinella spiralis. J. Immunol., 172, 1139–1145. 14707089 10.4049/jimmunol.172.2.1139

[4] Galli S.J. and Tsai M. (2012) IgE and mast cells in allergic disease. Nat. Med., 18, 693–704. 22561833 10.1038/nm.2755 PMC3597223

[5] Takhar P., Corrigan C.J., Smurthwaite L. et al. (2007) Class switch recombination to IgE in the bronchial mucosa of atopic and nonatopic patients with asthma. J. Allergy Clin. Immunol., 119, 213–218. 17208604 10.1016/j.jaci.2006.09.045

[6] Hamilton R.G. and Oppenheimer J. (2015) Serological IgE analyses in the diagnostic algorithm for allergic disease. J. Allergy Clin. Immunol. Pract., 3, 833–840. 26553612 10.1016/j.jaip.2015.08.016

[7] Hopp R.J., Bewtra A.K., Watt G.D. et al. (1984) Genetic analysis of allergic disease in twins. J. Allergy Clin. Immunol., 73, 265–270. 6538209 10.1016/s0091-6749(84)80018-4

[8] Lichtenstein P. and Svartengren M. (1997) Genes, environments, and sex: factors of importance in atopic diseases in 7–9-year-old Swedish twins. Allergy, 52, 1079–1086. 9404559 10.1111/j.1398-9995.1997.tb00179.x

[9] Hong X., Tsai H.-J. and Wang X. (2009) Genetics of food allergy. Curr. Opin. Pediatr., 21, 770–776. 19851108 10.1097/MOP.0b013e32833252dc PMC2892276

[10] Madore A.-M. and Laprise C. (2010) Immunological and genetic aspects of asthma and allergy. J. Asthma Allergy, 3, 107–121. 21437045 10.2147/JAA.S8970 PMC3047903

[11] Ober C. and Yao T.-C. (2011) The genetics of asthma and allergic disease: a 21st century perspective. Immunol. Rev., 242, 10–30. 21682736 10.1111/j.1600-065X.2011.01029.x PMC3151648

[12] Hong X. and Wang X. (2012) Early life precursors, epigenetics, and the development of food allergy. Semin. Immunopathol., 34, 655–669. 22777545 10.1007/s00281-012-0323-y PMC3439840

[13] Jiang P., Liu J., Yan X.-B. et al. (2009) Several interleukin-4 and interleukin-13 gene single nucleotide polymorphisms among Chinese asthmatic patients. Allergy Asthma Proc., 30, 413–418. 19772762 10.2500/aap.2009.30.3255

[14] Bønnelykke K., Sleiman P., Nielsen K. et al. (2014) A genome-wide association study identifies *CDHR3* as a susceptibility locus for early childhood asthma with severe exacerbations. Nat. Genet., 46, 51–55. 24241537 10.1038/ng.2830

[15] Gharagozlou M., Behniafard N., Amirzargar A.A. et al. (2015) Association between single nucleotide polymorphisms of the interleukin-4 gene and atopic dermatitis. Acta Dermatovenerol. Croat., 23, 96–100. 26228820

[16] Hong X., Hao K., Ladd-Acosta C. et al. (2015) Genome-wide association study identifies peanut allergy-specific loci and evidence of epigenetic mediation in US children. Nat. Commun., 6, 6304. 25710614 10.1038/ncomms7304 PMC4340086

[17] Tamari M., Tanaka S. and Hirota T. (2013) Genome-wide association studies of allergic diseases. Allergol. Int., 62, 21–28. 10.2332/allergolint.13-RAI-0539 28942987

[18] Bønnelykke K., Matheson M.C., Pers T.H. et al. (2013) Meta-analysis of genome-wide association studies identifies ten loci influencing allergic sensitization. Nat. Genet., 45, 902–906. 23817571 10.1038/ng.2694 PMC4922420

[19] Portelli M.A., Hodge E. and Sayers I. Genetic risk factors for the development of allergic disease identified by genome-wide association. Clin. Exp. Allergy, 45, 21–31. 24766371 10.1111/cea.12327 PMC4298800

[20] Yazdanbakhsh M., Kremsner P.G. and van Ree R. (2002) Allergy, parasites, and the hygiene hypothesis. Science, 296, 490–494. 11964470 10.1126/science.296.5567.490

[21] Fuleihan R., Ramesh N., Loh R. et al. (1993) Defective expression of the CD40 ligand in X chromosome-linked immunoglobulin deficiency with normal or elevated IgM. Proc. Natl. Acad. Sci. U. S. A., 90, 2170–2173. 7681587 10.1073/pnas.90.6.2170 PMC46047

[22] Speake C., Presnell S., Domico K. et al. (2015) An interactive web application for the dissemination of human systems immunology data. J. Transl. Med., 13, 196. 26088622 10.1186/s12967-015-0541-x PMC4474328

[23] Rinchai D., Boughorbel S., Presnell S. et al. (2016) A curated compendium of monocyte transcriptome datasets of relevance to human monocyte immunobiology research. F1000Res., 5, 291. 10.12688/f1000research.8182.2 PMC4856112 27158452

[24] Blazkova J., Boughorbel S., Presnell S. et al. (2016) A curated transcriptome dataset collection to investigate the immunobiology of HIV infection. F1000Res., 5, 327. 27134731 10.12688/f1000research.8204.1 PMC4838008

[25] Marr A.K., Boughorbel S., Presnell S. et al. (2016) A curated transcriptome dataset collection to investigate the development and differentiation of the human placenta and its associated pathologies. F1000Res., 5, 305. 27303626 10.12688/f1000research.8210.1 PMC4897750

[26] Rahman M., Boughorbel S., Presnell S. et al. (2016) A curated transcriptome dataset collection to investigate the functional programming of human hematopoietic cells in early life. F1000Res., 5, 414. 27347375 10.12688/f1000research.8375.1 PMC4916988

[27] Roelands J., Decock J., Boughorbel S. et al. (2017) A collection of annotated and harmonized human breast cancer transcriptome datasets including immunologic classification. F1000Res., 6, 296. 29527288 10.12688/f1000research.10960.1 PMC5820610

[28] Mackeh R., Boughorbel S., Chaussabel D. et al. (2017) A curated transcriptomic dataset collection relevant to embryonic development associated with in vitro fertilization in healthy individuals and patients with polycystic ovary syndrome. F1000Res., 6, 181. 28413616 10.12688/f1000research.10877.1 PMC5365227

[29] Rinchai D., Kewcharoenwong C., Kessler B. et al. (2016) Increased abundance of ADAM9 transcripts in the blood is associated with tissue damage. F1000Res, 4, 89. 10.12688/f1000research.6241.1 PMC5130078 27990250

[30] Rinchai D., Presnell S., Vidal M. et al. (2015) Blood interferon signatures putatively link lack of protection conferred by the RTS,S recombinant malaria vaccine to an antigen-specific IgE response. F1000Res, 4, 919. 28883910 10.12688/f1000research.7093.1 PMC5580375

[31] Chaussabel D. and Rinchai D. (2018) Using “collective omics data” for biomedical research training. Immunology, 155, 18–23. 29705995 10.1111/imm.12944 PMC6099165

[32] Kanehisa M., Goto S., Sato Y. et al. (2012) KEGG for integration and interpretation of large-scale molecular data sets. Nucleic Acids Res., 40, D109–D114. 22080510 10.1093/nar/gkr988 PMC3245020

[33] Giovannini-Chami L., Marcet B., Moreilhon C. et al. (2012) Distinct epithelial gene expression phenotypes in childhood respiratory allergy. Eur. Respir. J., 39, 1197–1205. 22005912 10.1183/09031936.00070511

[34] Holt P.G., Strickland D., Bosco A. et al. (2016) Distinguishing benign from pathologic TH2 immunity in atopic children. J. Allergy Clin. Immunol., 137, 379–387. 26518094 10.1016/j.jaci.2015.08.044

[35] Baos S., Calzada D., Cremades-Jimeno L. et al. (2018) Nonallergic asthma and its severity: biomarkers for its discrimination in peripheral samples. Front. Immunol., 9, 1416. 29977241 10.3389/fimmu.2018.01416 PMC6021512

[36] ELBadawy N.E., Abdel-Latif R.S. and El-Hady H.A. (2017) Association between SERPINB2 gene expression by real time PCR in respiratory epithelial cells and atopic bronchial asthma severity. Egypt J Immunol, 24, 165–181. 29120588

[37] Schroder W.A., Le T.T.T., Major L. et al. (2010) A physiological function of inflammation-associated SerpinB2 is regulation of adaptive immunity. J. Immunol., 184, 2663–2670. 20130210 10.4049/jimmunol.0902187

[38] Allen L., Scott J., Brand A. et al. (2014) Publishing: credit where credit is due. Nature, 508, 312–313. 24745070 10.1038/508312a

[39] Holland S.M., DeLeo F.R., Elloumi H.Z. et al. (2007) STAT3 mutations in the hyper-IgE syndrome. N. Engl. J. Med., 357, 1608–1619. 17881745 10.1056/NEJMoa073687

[40] Kosoy R., Agashe C., Grishin A. et al. (2016) Transcriptional profiling of egg allergy and relationship to disease phenotype. PLoS ONE, 11, e0163831. 27788149 10.1371/journal.pone.0163831 PMC5082817

