# Supplementary material for: A curated collection of transcriptome datasets to investigate the molecular mechanisms of immunoglobulin E-mediated atopic diseases
Source: Database (Oxford). 2019 Jul 10;2019:baz066. doi: 10.1093/database/baz066 (PMC6616200; doi:10.1093/database/baz066)

**SUPPLEMENTAL INFORMATION**

**Supplement Information 1.** Data Browsing and Visualization Interface

By default, the available data in the curation are listed and sorted by their titles. However, the table can also be sorted by other column headings such as Platform, Species, Disease, Sample Source, and Sample Count. If available, the G and M icons in the table direct users to the original dataset entry on NCBI’s GEO and its associated article on PubMed, respectively. Filter options on the left side of the page can be applied to produce a list of datasets that meet the specific criteria (i.e. Disease, Sample Source, and Platform). For example, filtering for “Healthy” under Disease will return 7 datasets.

To browse individual datasets, simply click on the dataset title of interest under Sample Set. The genes are automatically ranked in a descending order based on their absolute FC for the default pairwise comparison. The direction of change is indicated by the colour and direction of the triangles (red/up and blue/down for up and down-regulated, respectively). Information about the selected Gene, Study, and Sample are displayed on the panel below the dataset title. Both the gene list and information panels can be hidden to maximize the graphical display.

The graphical display can be changed to box plot from the default bar plot using the
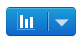
dropdown menu. The default numerator and the denominator group for the FC calculation is indicated on the X-axis, by the red and blue dot, respectively. In datasets with more than two groups, specific comparison can be changed via the “Rank Lists” dropdown menu, where all the available pairwise groups can be selected from “Rank Lists”. The default pairwise comparison is indicated by the prefix “+” in this dropdown list. The graph can be further customized via “Tools” (upper right-and corner), followed by the various options offered by “Chart Options”.

The graphical output can be downloaded as is directly from GXB by clicking
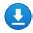
. As well, the associated metadata and expression value of the selected gene of interest can be obtained as a csv file by clicking
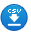
. Additional sample information in csv formats can also be downloaded using the dropdown menu found in the Downloads panel. The Annotations for Group Set include the metadata for the entire dataset and the Annotations for Group allows select group metadata download.

The interface can be queried for studies in which a gene of interest is differentially expressed between two groups in two options. In the first option, Gene Symbol of interest can be entered in the search bar under Significant Genes in the Data Browser page. A dropdown menu of available Gene Symbols will appear once the gene symbol is entered. The default threshold for FC cut-off is set at 2.0 but can be adjusted using the sliding bar. For example, a search of CD40 at a cut-off of 2.0 FC will return three datasets (GSE56681, GSE44956, and GSE70050). The default pair-wise rank comparison for each dataset and the associated FC for the gene of interest is shown below the dataset title. Clicking on the Sample Set title will allow browsing of the individual dataset, where FC values for the associated probes of the gene symbol of interests will be displayed both numerically and graphically. As previously mentioned, the specific group comparison can be changed by the user via the Rank Lists dropdown function. Once the new comparison group is selected and the Reset Rank List is clicked, new FC values for CD40 will be displayed. The second option displays the gene of interest in all datasets and the users can scroll down the individual dataset in the same page display. To do so, simply click on any dataset and click select the gene of interests. Then from the “Tools” option in the upper right-hand corner, click on “Cross Project View”. However, this option does not allow pairwise comparison, thus no FC values will be found.

**Supplement Information 2.** Calculation of FC Expression.

GXB uses the geometric means of replicates of each sample and calculates FC via the difference (for data in log2 scale) or the ratio (for data in linear scale) of the means. The data displayed in GXB are linear scale FC. The following example illustrates the calculation:

**GSE ID:** GSE54336

**Type of file:** *.soft file (The data deposited by the contributor were analyzed with Partek Genomic Suite 6.6 using Affymetrix default analysis settings, quantile normalization and RMA background correction)

**Number of samples:** 6

**Number of groups:** 3 (G1TEPP, G1V, and Mock)

**Groups compared** (for this example)**:** G1TEPP and G1V

**Gene symbol:** DUSP2

**Probe set ID:** 204794_at

**Sample Information:**

GSM1313408 A2EN cells_Chlamydia G1TEPP_4h_rep1

GSM1313409 A2EN cells_Chlamydia G1TEPP_4h_rep2

GSM1313410 A2EN cells_Chlamydia G1V_4h_rep1

GSM1313411 A2EN cells_Chlamydia G1V_4h_rep2

GSM1313412 A2EN cells_Mock infected_repl1

GSM1313413 A2EN cells_Mock infected_repl2

**Condition 1: G1TEPP (signal value in log_2_ scale)**

GSM1313408: 6.31727

GSM1313409: 6.26554

Geometric Mean = sqrt (6.31727 * 6.26554)

🡪 Geometric Mean = 6.29135183214

**Condition 2: G1V (signal value in log_2_ scale)**

GSM1313410: 6.93182

GSM1313411: 6.88052

Geometric Mean = sqrt (6.93182 * 6.88052)

🡪 Geometric Mean = 6.90612236689

**Calculation of FC expression (G1TEPP/G1V) and transformation to linear scale FC**:

log_2_ FC = Condition 1 - Condition 2

log_2_ FC = 6.29135183214 - 6.90612236689 = -0.61

linear FC = Antilog (-0.61) = 2^(-0.61)^ = 0.65

Mathematical transformation when linear FC is less than 1: -1/(FC)

🡪 -1/0.65 = -1.52 (i.e. down regulation in linear scale)

**Reference**:

The dataset used in this example can be accessed via these links:

GXB: <http://cd2k.gxbsidra.org/dm3/geneBrowser/show/4000099>

GEO2R: <https://www.ncbi.nlm.nih.gov/geo/geo2r/?acc=GSE54336>

**Supplement Information 3. T-test for Pairwise Group Comparison**.

Gene expression data from dataset of interest can be downloaded individually from the GXB to perform statistical analyses such as the T-test. The following describes the steps for a T-test calculation between two groups of interest. GSE19190 is used as an example to calculate p-value for SERPINB2 expression between Rhinitis and Healthy controls:

1. Navigate to the GXB web browser application and click on “Distinct epithelial gene expression phenotypes in childhood respiratory allergy - GSE19190 - Disease State” (<http://ige.gxbsidra.org/dm3/geneBrowser/show/4000082>).
2. In the search box on the top left pane, input the “Gene Symbol” as “**SERPINB2**” and press enter (<http://ige.gxbsidra.org/dm3/miniURL/view/Lr>).
3. Click on “Download Sample data as csv” button present on the top right pane of the plot.
4. Open the downloaded *.csv file. Note: It is recommended to open it in MS excel format for the ease of computation.
5. In excel, navigate to the menu bar, Formulas > Insert Functions > Search for **TTEST** in the search box and double click on it.
6. In the **Array1**, select all the first condition (Rhinitis) expression values and in the **Array2**, select the second condition (Healthy) expression values. After this, input values **Tails = 2** and **Type = 3**. Click on Done.
7. This will output a p-value **0.015437451**.

**SUPPLEMENT TABLES AND FIGURES**

**Suppl. Table 1.** Top 50 differently expressed genes in datasets GSE70760 and GSE19190 as represented on GXB.

**
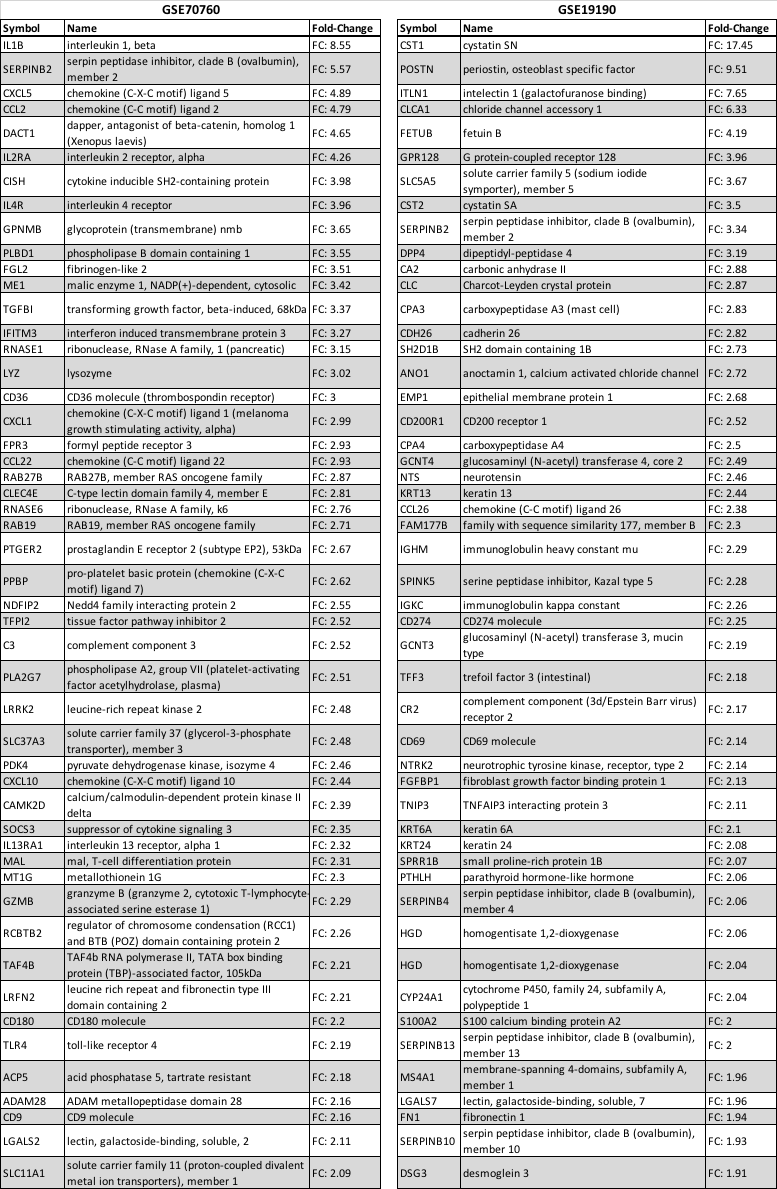
**

SUPPL. FIGURE 3


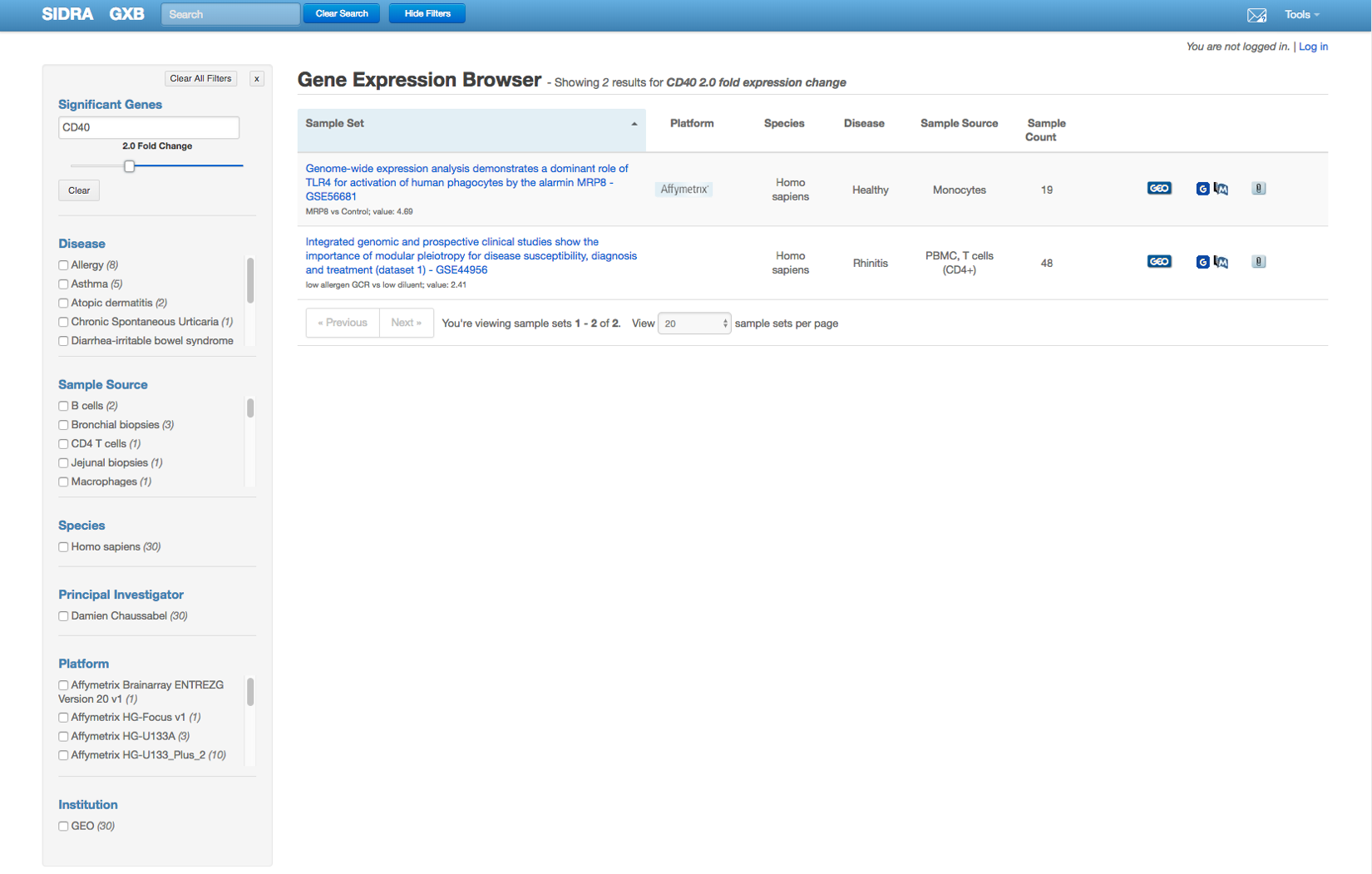

Supplement: SUPPLEMENTAL_INFORMATION_baz066 [file supplemental_information_baz066.docx]
